# Preoperative risk factors for early postoperative bleeding after Roux-en-Y gastric bypass surgery: a systematic review and meta-analysis

**DOI:** 10.1007/s00423-024-03346-4

**Published:** 2024-05-22

**Authors:** Hugo Santos-Sousa, Filipe Amorim-Cruz, Jorge Nogueiro, Alexandre Silva, Inês Amorim-Cruz, Rui Ferreira-Santos, Raquel Bouça-Machado, André Pereira, Fernando Resende, André Costa-Pinho, John Preto, Eduardo Lima-da-Costa, Elisabete Barbosa, Silvestre Carneiro, Bernardo Sousa-Pinto

**Affiliations:** 1 Obesity Integrated Responsability Unit (CRI-O), São João University Medical Center, Porto, Portugal; 2 Faculty of Medicine, University of Porto, Porto, 4200 - 319 Portugal; 3 Surgery Department, São João University Medical Center, Porto, Portugal; 4 Instituto de Medicina Molecular João Lobo Antunes, Lisbon, Portugal; 5 Department of Community Medicine, Information and Health Decision Sciences, Faculty of Medicine, MEDCIDS, University of Porto, Porto, Portugal; 6 CINTESIS – Center for Health Technology and Services Research, University of Porto, Porto, Portugal

**Keywords:** Bariatric surgery, Obesity, Roux-en-Y gastric bypass, Risk factors, Hemorrhage

## Abstract

**Purpose:**

Although bariatric surgery is an effective intervention for obesity, it comes with risks such as early postoperative bleeding (EPB). Identifying preoperative risk factors for this complication can help patients’ risk stratification and optimization. We performed a systematic review and meta-analysis to find predictors for early postoperative bleeding after Roux-en-Y gastric bypass (RYGB).

**Methods:**

We conducted a systematic review, searching PubMed, Cochrane Library, and Web of Science until November 2023. We performed a random-effects meta-analysis to explore preoperative risk factors associated with early postoperative bleeding after RYGB. Sources of heterogeneity were explored by leave-one-out analyses.

**Results:**

23 studies were included, comprising 232,488 patients. Male gender (meta-analytical RR = 1.42, 95%CI = 1.21–1.66, I^2^ = 18%, Q Cochran test p-value = 0.29) and revisional surgery (meta-analytical RR = 1.35, 95%CI = 1.12–1.62, I^2^ = 22%, Q Cochran test *p* = 0.21) were associated with higher risk of EPB. On average, patients with EPB were older than the remainder (MD for the mean age = 2.82 years, 95%CI = 0.97–4.67, I^2^ = 0.00%, Q Cochran test *p* = 0.46). Except for hypertension (meta-analytical RR = 1.33, 95%CI = 1.02–1.73, I^2^ = 66%, Q Cochran test *p* < 0.0001), comorbidities were not associated with a higher risk of EPB.

**Conclusion:**

Preoperative risk factors, including age, gender, hypertension, and revisional bariatric surgery, are associated with early postoperative bleeding after RYGB. Further primary studies, with higher methodological quality, are required to detail more risk factors.

**Supplementary Information:**

The online version contains supplementary material available at 10.1007/s00423-024-03346-4.

## Introduction

Obesity is a complex multifactorial disease, whose prevalence has been increasing steadily in the last decades [1]. Bariatric surgery has been identified as the most effective treatment for clinically severe obesity, resulting in substantial and sustained weight loss and improvement in obesity-related comorbidities [2]. Considering its metabolic impact, Roux-en-Y gastric bypass (RYGB) remains one of the most performed metabolic procedures [3]. Although the safety profile of RYGB has improved over the past few decades [4], as the number of procedures performed continues to increase, it is important to acknowledge that the risk of major early complications is not inexistent.

Postoperative bleeding is one of the most common complications in the early postoperative period after RYGB [5], leading to longer hospital stays, higher reoperation rates, and higher mortality rates. Understanding the risk factors associated with early postoperative bleeding might be important for better preoperative optimization of modifiable risk factors and individual risk stratification aiming for closer surveillance in the early postoperative period. During RYGB, intraoperative and technical risk factors associated with EPB had already been systematically studied – for instance, performing a mechanic gastrojejunal anastomosis with a circular stapler instead of a hand-sewn and not reinforcing the staple line increases the risk of bleeding [6, 7]. On the other hand, in contrast to intraoperative risk factors, there is no systematic evidence of preoperative clinical factors, even though there is growing evidence that patient illness severity, demographic characteristics, and specific preoperative comorbidities may increase the likelihood of postoperative bleeding. Therefore, in this systematic review and meta-analysis, we aimed to identify preoperative predictors of early postoperative bleeding in patients undergoing RYGB.

## Materials and methods

This systematic review with meta-analysis follows the Preferred Reporting Items for Systematic reviews and Meta-analyses (PRISMA) statement guidelines and the recommendations of the Cochrane Handbook for Systematic Reviews [8, 9].

### Literature search and eligibility criteria

We searched MEDLINE, Scopus, and Web of Science from inception to November 2023 using a pre-defined search strategy (Supplementary Table 1). Through Tripdatabase (https://www.tripdatabase.com), this search was supplemented by gray literature search as well as by hand-searching references of primary studies that were included. No restrictions were set regarding language or publication year.

Original studies were included if (i) RYGB was an obesity treatment for patients with a BMI ≥ 40 kg/m^2^ or BMI ≥ 35 kg/m^2^ with weight-related comorbidities, and (ii) incidence of early postoperative bleeding was reported, in combination with evaluation of preoperative risk factors, comparing patients with and without EPB. Early postoperative bleeding was defined as bleeding occurring in a period of up to 30 days after RYGB surgery. More details on the adopted inclusion and exclusion criteria are available in Supplementary Table 2.

### Study selection and data extraction

After removing duplicates, each study was independently assessed by two reviewers (H.S.S and F.C), first by title and abstract screening, and then by full-text reading. Any disagreements were resolved by consulting a third reviewer to reach a final decision.

Two reviewers independently extracted data from selected studies using a predefined spreadsheet purposely built for this systematic review. For each primary study, the following information was retrieved: authors’ identification, the year of publication, country, study design, timing and surgical technique (ORYGB, LRYGB, RRYGB), frequency of co-morbidities, total number of patients with EPB after RYGB, and number of patients with (and without) EPB according to the presence or absence of potential preoperative risk factors.

### Quality of reporting analysis

The quality of primary studies was independently assessed by two reviewers (H.S.S and F.C) using the Methodological Index for Non-Randomized Studies (MINORS) [10]. To reach a consensus, divergent opinions regarding quality assessment were discussed with a third reviewer. This tool consists of a form with 12 items - the first eight being specifically for non-comparative studies (related to the research question, study population, exposure, outcome, blinding, follow-up, and statistical analysis) [10]. For this review and meta-analysis, a score of < 8 was considered to be poor quality, 9–14 moderate quality, and 15–16 good quality for noncomparative studies. Cutoff points were < 14, 15–22, and 23–24, respectively, for comparative studies [10]. 

### Synthesis of results

We performed a random-effects meta-analysis using the DerSimonian-Laird method, computing pooled meta-analytical values for the association between each potential preoperative risk factor and postoperative bleeding. We calculated pooled risk ratios (RR) for categorical variables and pooled mean differences (MD) for continuous variables. If a risk factor was pooled in two or more primary studies, a meta-analysis was performed, assuring that if only two primary studies were included, they could be meaningfully pooled and provided sufficiently ‘similar’ results. Heterogeneity was assessed using the Q-Cochran test statistic p-value and the *I*^2^ statistic. For the Q-Cochran test statistic, p-values < 0.10 were considered to indicate significant heterogeneity. For the *I*^2^ statistic, we considered 0% to correspond to the absence of detected heterogeneity, 0–10% to be low heterogeneity, 10–50% to be moderate heterogeneity, and above 50% to be high heterogeneity [8]. Heterogeneity sources were explored through leave-one-out sensitivity analyses. Statistical analysis was performed using R statistical software (version 4.3.2), with the use of the ‘‘meta’’ [11] and ‘‘gtsummary’’ packages [12].

## Results

### Study selection and characteristics

**Fig. 1:**
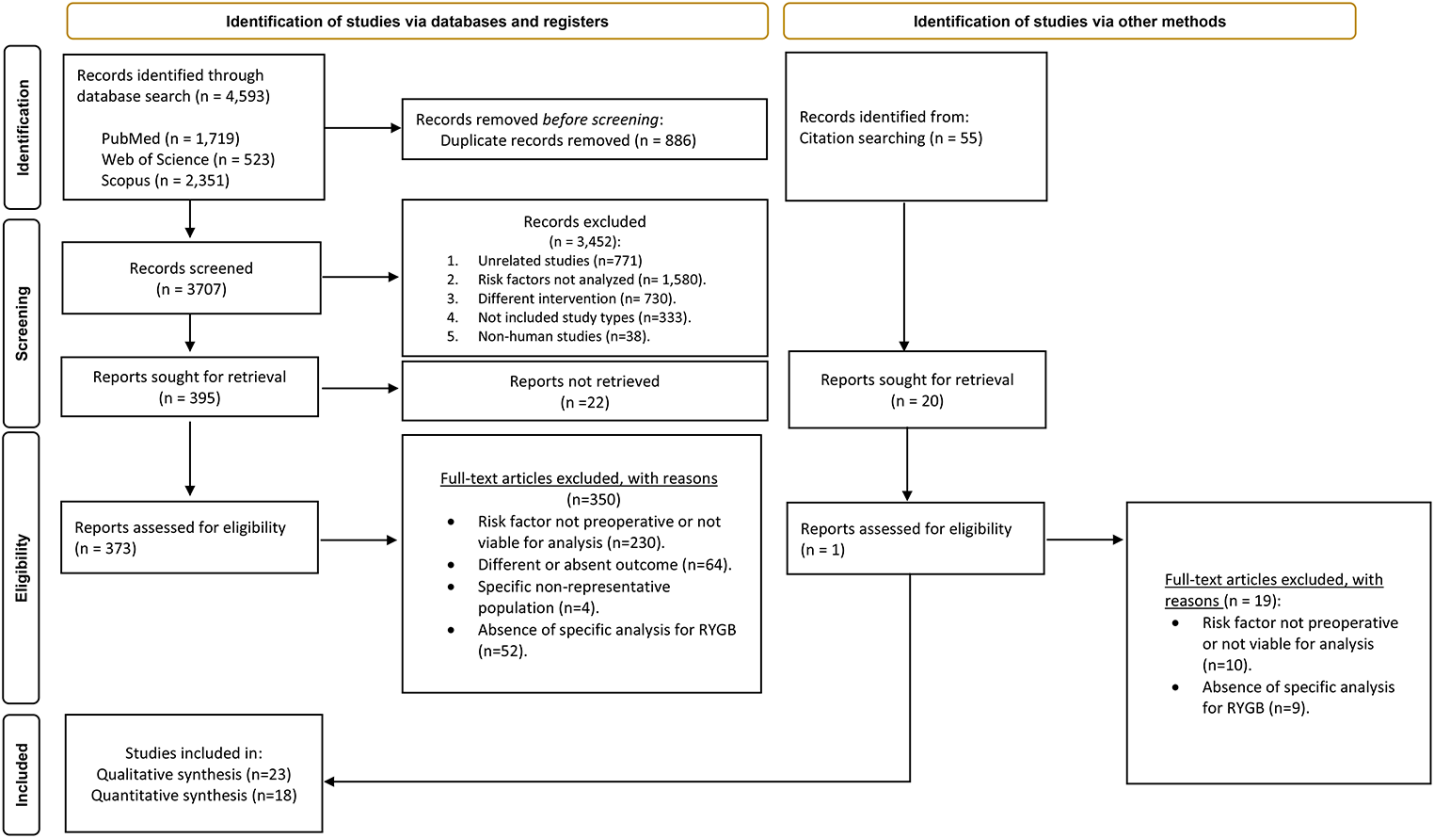
PRISMA flow chart of the study

The electronic literature search yielded 4,593 articles, of which 886 were duplicates. After excluding 3,474 records in the screening phase, 373 articles were fully read, of which a total of 22 were included in the systematic review (Fig. 1). Hand-searching resulted in 55 additional articles, of which 1 was included. In total, 23 articles were included [13–35].

A summary of included studies is presented in Table 1. A more detailed presentation of the studies’ characteristics is reported in Supplementary Table 3. Studies were published between 2009 and 2023, with a cumulative sample size of 232,488 patients. Most studies [13–18, 20–22, 24–33, 35] were retrospective cohort studies (*n* = 21, 91.30%), with the remaining being prospective observational studies [19, 23]. The mean participants’ age was 45.2 years (SD = 10.8 years) with a female predominance (79.30%). Laparoscopic Roux-en-Y gastric bypass (LRYGB) was the most performed RYGB type (*n* = 211,723, 91.10%) and 204,952 (88.0%) of patients were submitted to a primary surgery.

**Table 1:** Studies general characteristics

Study	Yr	Study Design	Sample Size	Follow up (month)	Patients with EPB (*n*)	Presence of Risk Factor in Bleeding Patients (*n*)	Presence of Risk Factor in non-bleeding Patients (*n*)	Average age in patients with bleeding (years)	Average age in patients without bleeding (years)
Bakhos C.	2009	RS	132	1	33	Male gender: 7	Male gender: 18	47.5 ± 8.7	42.8 ± 10.8
						Superobesity: 15	Superobesity: 48		
						DVT P. Heparin: 16	TVP P. Heparin: 69		
						DVT P. Enoxaparin: 15	TVP P. Enoxaparin: 25		
Dick A.	2010	PS	783	1	26	Male gender: 5	Male gender: 135	47 ± 12	44 ± 11.6
						Diabetes mellitus: 7	Diabetes mellitus: 288		
						Hypertension: 13	Hypertension:497		
						Dyslipidemia: 6	Dyslipidemia: 301		
						OSA: 9	OSA: 261		
						GERD: 9	GERD: 324		
						Revisional Surgery: 0	Revisional Surgery: 13		
Rabl C.	2011	RS	722	20.9	19	Male gender: 1	Male gender: 134	44.9 ± 9.4	44.8 ± 10.6
						Diabetes mellitus: 11	Diabetes mellitus: 227		
						Hypertension: 12	Hypertension: 439		
						OSA: 5	OSA: 250		
Deylgat B.	2012	RS	724	45	11	Revisional Surgery: 3	Revisional Surgery: 69	NR	NR
Slegtenhorst B.R.	2013	RS	283	NR	1	Revisional Surgery: 0	Revisional Surgery: 66	NR	NR
Stenberg E.	2014	RS	25,038	1	522	Male gender: 174	Male gender: 5,874	NR	NR
						Diabetes mellitus: 120	Diabetes mellitus: 3640		
						Hypertension: 194	Hypertension: 6127		
						Dyslipidemia: 88	Dyslipidemia: 2452		
						OSA: 72	OSA: 2373		
						Cardiovascular Disease: 194	Cardiovascular Disease: 6127		
Sadot E.	2015	RS	126	72	8	Revisional Surgery: 3	Revisional Surgery: 41	NR	NR
Coblijn U.K.	2016	RS	1667	96	43	Revisional Surgery: 10	Revisional Surgery: 298	NR	NR
Ramly E.P.	2016	RS	66,078	60	817	Revisional Surgery: 17	Revisional Surgery: 1195	NR	NR
Al-Kurd A.	2018	RS	322	12	12	Revisional Surgery: 4	Revisional Surgery: 2	NR	NR
Zafar S.N.	2018	RS	43,280	1	652	Age ≥ 60 years: 114	Age ≥ 60 years: 5714	NR	NR
						Male gender: 156	Male gender: 8483		
						Superobesity: 171	Superobesity: 11,200		
						Diabetes mellitus: 396	Diabetes mellitus: 28,134		
						Hypertension: 398	Hypertension: 22,623		
						Dyslipidemia: 228	Dyslipidemia: 12,446		
						OSA: 287	OSA: 17,676		
						GERD: 277	GERD: 16,221		
						Cardiovascular Disease: 70	Cardiovascular Disease: 2127		
						Revisional Surgery: 90	Revisional Surgery: 3591		
Axer S.	2019	RS	47,850	60	969	Revisional Surgery: 52	Revisional Surgery: 917	NR	NR
Nasser H.	2019	RS	12,442	1	221	Revisional Surgery: 221	Revisional Surgery: 12,221	NR	NR
León-Ballesteros G.P	2020	RS	849	12	16	Age ≥ 60 years: 0	Age ≥ 60 years: 57	NR	NR
Poublon N.	2020	RS	306	36	8	Revisional Surgery: 8	Revisional Surgery: 298	NR	NR
Turchi M.J	2020	RS	582	60	22	Age ≥ 60 years: 2	Age ≥ 60 years: 51	NR	NR
Jung J.J	2021	RS	14,896	1	226	Revisional Surgery: 25	Revisional Surgery: 1115	NR	NR
Joel S. Frieder	2021	RS	321	24	3	Age ≥ 60 years: 3	Age ≥ 60 years: 318	NR	NR
Odovic M.	2022	RS	2639	1	72	Male gender: 27	Male gender: 607	44.9 ± 12.4	42.1 ± 12.4
						Superobesity: 10	Superobesity: 449		
						Diabetes mellitus: 47	Diabetes mellitus: 1715		
						Hypertension: 43	Hypertension: 1182		
						Dyslipidemia: 44	Dyslipidemia: 1734		
						OSA: 39	OSA: 1437		
						Revisional Surgery: 5	Revisional Surgery: 146		
Campo-Betancourth C.F	2022	RS	628	1	51	Revisional Surgery: 1	Revisional Surgery: 50	NR	NR
Pereira A.	2022	RS	340	1	14	Male gender: 3	Male gender: 35	47	46
						Diabetes mellitus: 4	Diabetes mellitus: 91		
						Hypertension: 5	Hypertension: 153		
						Dyslipidemia: 5	Dyslipidemia: 149		
						OSA: 1	OSA: 57		
						GERD: 5	GERD: 58		
						Revisional Surgery: 5	Revisional Surgery: 78		
O’Laughlin M.	2023	PS	11,824	1	66	Revisional Surgery: 33	Revisional Surgery: 5879	NR	NR
Reiter A.J	2023	RS	656	120	22	DVT P. Heparin: 7	DVT P. Heparin: 379	NR	NR
						DVT P.Enoxaparin: 15	DVT P. Enoxaparin: 255		

Yr: Year of publication, n: number of patients, EPB: Early (<30 days) Postoperative Bleeding, SD: Standard Deviation; Cardiovascular Disease includes Ischemic heart disease (Acute coronary syndrome, Stable coronary artery disease, history of arterial revascularization), Stroke or Transient Ischemic Attack, Peripheral artery disease, Aortic aneurysm; GERD: Gastroesophageal reflux disease, OSA: Obstructive sleep apnea; DVT P. – Deep Vein Thrombosis Prophylaxis; NR: Not reported

### Preoperative risk factors for early postoperative bleeding after RYGB

**Fig. 2:**
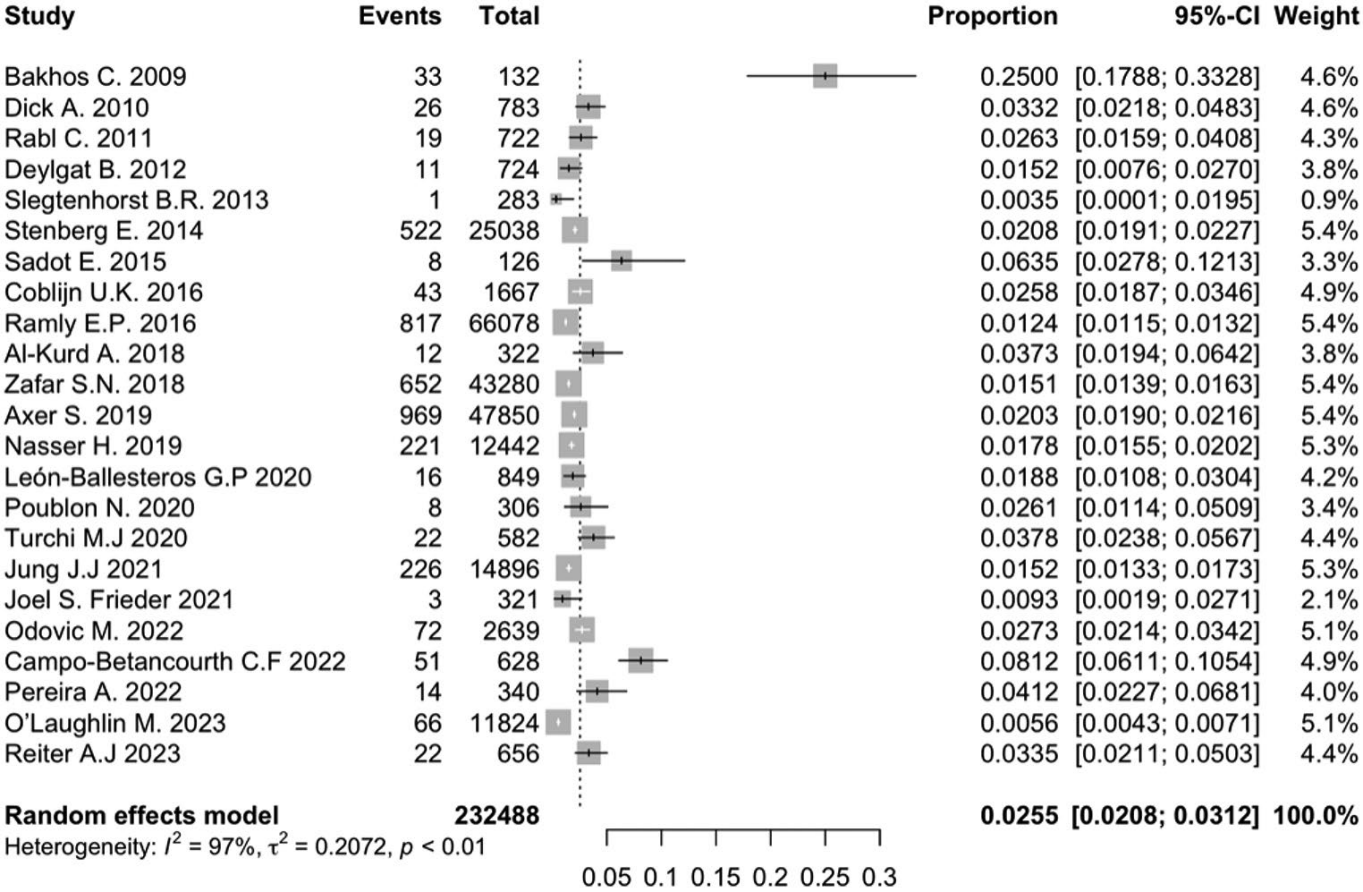
Forest plot representing the cumulative incidence of postoperative bleeding after RYGB in the first 30 days

Our meta-analysis showed that the cumulative incidence of early postoperative bleeding after RYGB was 2.60% (95% CI 2.1–3.1%), I^2^ = 97%; Q Cochran test p-value < 0.01; Fig. 2). We were able to do a meta-analysis on the following risk factors (Table 2): average participants’ age, gender, patients with a BMI > 50 kg/m^2^, patients’ comorbidities (diabetes mellitus, hypertension, dyslipidemia, obstructive sleep apnea (OSA), gastroesophageal reflux disease (GERD)), prophylaxis for thromboembolism (heparin versus enoxaparin) and previous bariatric surgery requiring revisional RYGB (rRYGB). Male gender (meta-analytical RR = 1.42, 95%CI = 1.21–1.66, I^2^ = 18%, Q Cochran test p-value = 0.29; Supplementary Fig. 2) and revisional RYGB (meta-analytical RR = 1.35, 95%CI = 1.12–1.62, I^2^ = 22%, Q Cochran test *p* = 0.21; Supplementary Fig. 9) were significantly associated with higher risk of early postoperative bleeding after RYGB. For gender, in leave-one-out sensitivity analysis, omitting Rabl et al. [28], there was a decrease from 18 to 0% in I^2^ (Supplementary Table 6). On average, patients with EPB were older (MD for the mean participants’ age = 2.82 years, 95%CI = 0.97–4.67, I^2^ = 0.00%, Q Cochran test *p* = 0.46; Supplementary Fig. 1). Compared with patients with BMI < 50 kg/m^2^, patients with BMI > 50 kg/m^2^ had no statistically significant increased risk of EPB after RYGB (meta-analytical RR = 0.97, 95%CI = 0.83–1.15, I^2^ = 0%, Q Cochran test *p* = 0.73; Supplementary Fig. 3). Comorbidities were not significantly associated with increased risk of EPB, except for hypertension (meta-analytical RR = 1.33, 95%CI = 1.02–1.73, I^2^ = 66%, Q Cochran test *p* < 0.0001; Supplementary Fig. 5). Even though a leave-one-out sensitivity analysis was performed, we observed that no primary study was a significant source of heterogeneity for diabetes mellitus(Supplementary Table 8), dyslipidemia (Supplementary Table 10), and GERD (Supplementary Table 12). For hypertension, in leave-one-out sensitivity analysis, omitting Dick et al. [19], there was a decrease from 66 to 46.4% in I^2^ and a stronger association was found (meta-analytical RR = 1.50, 95%CI = 1.23–1.83, Supplementary Table 9). Preoperative administration of unfractionated heparin was associated with a lower risk of EPB after RYGB compared to enoxaparin (RR = 0.37, 95%CI = 0.23–0.58, I^2^ = 0.00%, Q Cochran test *p* = 0.68; Supplementary Fig. 10).

**Table 2:** Preoperative risk factors for early postoperative bleeding

Preoperative Risk Factor for EPB	Studies (*n*)	Total Patients (*n*)	Patients with EPB (*n*)	Random effects model	I^2^	Q Cochran test *p*
Age	4	4616	164	MD = 2.82, 95%CI = 0.97–4.67	0%	0.46
Gender	7	72,934	373	RR = 1.42, 95%CI = 1.21–1.66	18%	0.29
BMI > 50 kg/m^2^	3	45,825	754	RR = 0.97, 95%CI = 0.83–1.15	0%	0.73
Diabetes Mellitus	6	72,802	585	RR = 1.13, 95%CI = 0.72–1.76	87%	< 0.01
Arterial Hypertension	6	72,802	665	RR = 1.33, 95%CI = 1.02–1.73	66%	< 0.01
Dyslipidemia	5	72,080	371	RR = 1.07, 95%CI = 0.74–1.55	79%	< 0.01
Obstructive Sleep Apnea	6	72,802	413	RR = 1.15, 95%CI = 0.94–1.40	32%	0.2
GERD	3	44,403	692	RR = 1.19, 95%CI = 0.76–1.85	40%	0.19
Revisional RYGB Surgery	14	191,440	248	RR = 1.35, 95%CI = 1.12–1.62	22%	0.21
Prophylaxis for thromboembolism	2	728	53	RR = 0.37, 95%CI = 0.23–0.58	0%	0.68

n: number of patients, EPB: Early (<30 days) Postoperative Bleeding, MD: Mean Difference, RR: Risk Ratio, RYGB: Roux-en-Y gastric bypass, BMI: Body Mass Index, GERD: Gastroesophageal reflux disease

### Quality of reporting analysis

The results of the MINORS risk of bias assessments for included primary studies are presented in Supplementary Table 4. Most studies (*n* = 17, 73.9%) were classified as having a moderate quality study, with the remaining [16–18, 20, 22, 27] being poor quality studies. Most studies (*n* = 19, 82.60%) did not mention an established protocol before the beginning of the study. A total of 8 (34.8%) studies had a reported loss to follow-up up less than 5%. The main difference between moderate and poor quality studies was reporting baseline equivalence of the groups and/or adequate statistical analysis (with the calculation of confidence intervals or relative risk).

## Discussion

In this study, we systematically reviewed the literature on preoperative risk factors associated with EPB, having identified five factors that were significantly associated with a higher risk of this complication - older age, male gender, hypertension, revisional RYGB, and preoperative administration of enoxaparin.

Gastrointestinal bleeding after gastric bypass surgery, although rare (with an estimated incidence of 1.1–4%), is one of the most common early complications after RYGB. It most commonly occurs at the anastomotic staple lines and presents within 12–24 h after surgery [36]. It is usually self-limited, but it can present as a life-threatening condition. It is crucial to identify the risk factors for early bleeding after bariatric surgery to prevent and manage this complication effectively. Surgical teams, being aware of this information, could take the necessary precautions and closely monitor patients who may be at higher risk of EPB, ensuring a safe and successful postoperative period.

Our meta-analysis showed that the risk of EPB was significantly associated with being of the male gender, which is in line with the recent literature [37–39]. Male gender has been associated with a higher incidence of overall postoperative complications or mortality after bariatric surgery, specifically, in RYGB. This could be explained by the more complicated male anatomy, such as the central distribution of adipose tissue, a higher prevalence of massive steatosis, and an increased liver size [37, 40]. In our meta-analysis, four retrospective studies [15, 19, 24, 25, 28] analyzed the effect of age, as a continuous variable, demonstrating that, on average, patients with EPB tend to be older, possibly due to the increased pre- and post-operative comorbidities. For older patients, Xu et al. [41] and Kermansaravi et al. [42] suggest that sleeve gastrectomy is safer than RYGB, despite RYGB being more effective in reducing obesity-related diseases and weight loss outcomes.

Revisional RYGB surgery is technically challenging due to altered anatomy and the presence of adhesions. Thus, it has a higher complication rate, including postoperative bleeding [22]. In our meta-analysis, this preoperative risk factor was the most well-studied variable, being reported by 14 primary studies [13, 14, 16–19, 21, 23–25, 29, 31, 32, 35]. In line with the literature, we found a significant association between revisional RYGB surgery and early postoperative bleeding.

Prophylaxis for thromboembolism after bariatric surgery is a delicate balance between reducing venous thromboembolism rates (0.3-1.5%) [30] and avoiding bleeding complications after the surgery. Our study found that the use of unfractionated heparin for thromboembolism chemoprophylaxis before surgery is associated with a lower risk of EPB compared to using enoxaparin. Given the low number of studies [15, 30] on this matter, this result should be interpreted with caution, and more primary studies are needed to draw conclusive evidence.

In our meta-analysis, except for hypertension, we did not find traditional hemorrhage-associated comorbidities to be associated with a higher risk of bleeding. Hypertension had previously been shown to be a risk factor for mortality after RYGB, due to an abnormal response to injury [40]. In our study, hypertension was found to be statistically significantly associated with an increased risk of EPB, with a stronger association when Dick et al. [19], inducing significant heterogeneity, was omitted in leave-one-out sensitivity analyses. We found a trend between OSA and postoperative bleeding, but it did not reach statistical significance. Neither diabetes mellitus, dyslipidemia nor GERD were shown to be preoperative risk factors for early postoperative bleeding. The role of diabetes as a risk factor for postoperative complications after RYGB is not completely established. Diabetes is associated with a pro-inflammatory state and slower wound healing, being a risk factor for cardiovascular, respiratory, infectious, and renal complications [43]. In our meta-analyses, the included studies with the largest samples reached opposite conclusions about the role of diabetes in early postoperative bleeding [33, 35]. Our findings should be interpreted with caution and require further investigation. Although a higher BMI has been identified as a risk factor for morbidity and mortality after bariatric surgery [44], our study did not find any association between a BMI greater than 50 kg/m^2^ and bleeding risk. However, it is important to interpret this result with caution since the effect of BMI was not possible to be analyzed as a continuous variable; and the literature [45] shows that extremes of BMI are associated with increased risk of early postoperative bleeding.

This systematic review has some limitations worth noting. An important limitation concerns the severe heterogeneity found, mainly in comorbidities’ patients’ risk factors, mirroring not only the nature of this meta-analysis (i.e. a quantitative synthesis of risk factors of a rare and unreported event such as early postoperative bleeding after RYGB) but also the differences in eligibility criteria across primary studies. To explore possible sources of heterogeneity, we performed leave-one-out analyses, although severe heterogeneity did not cease. Second, in part due to the characteristics of primary studies, during the selection process, 230 studies were excluded for not reporting preoperative risk factors for EPB; and, during data extraction, some variables, such as total weight loss and pre- and pos-bariatric BMI, were not computed due to lack of information. Finally, the assessment of the quality of some studies is hampered by the lack of follow-up of less than 5% and unreported criteria on the methods of participant selection or data collection. These limitations raise the need for subsequent studies to be performed with rigorous study designs and validated methods.

There are also strengths in our study. To the best of our knowledge, this is the first systematic review and meta-analysis to examine preoperative risk factors associated with early postoperative bleeding. Hence, we believe that the generated information is relevant to the field and to conduct future studies. Second, we performed a comprehensive search, encompassing three different electronic bibliographic databases and not applying exclusion criteria based on the date or the language of publication.

In conclusion, our results suggest that, after RYGB, patients with some preoperative risk factors, including age, gender, hypertension, revisional bariatric surgery, and preoperative administration of enoxaparin may need to be more judiciously monitored for early bleeding, as they are at increased risk of developing such event. Preoperative optimization of hemorrhagic risk factors is an essential step in the preoperative assessment of these patients. Further research on more preoperative risk factors could allow the building of a clinical tool to predict early postoperative bleeding risk.

### Electronic supplementary material

Below is the link to the electronic supplementary material.

Supplementary Material 1

## Data Availability

No datasets were generated or analysed during the current study.
