# Supplementary material for: Preoperative risk factors for early postoperative bleeding after Roux-en-Y gastric bypass surgery: a systematic review and meta-analysis
Source: Langenbecks Arch Surg. 2024 May 22;409(1):163. doi: 10.1007/s00423-024-03346-4 (PMC11111548; doi:10.1007/s00423-024-03346-4)

**Supplementary Material**

**Supplementary Table 1** – Search terms and queries

| MEDLINE/PubMed | (Postoperative AND (Bleeding OR Hemorrhage OR haemorrhage OR "blood loss")) AND (("Gastric bypass"[Title/Abstract] OR "Bariatric bypass surgery"[Title/Abstract] OR "Roux-en-Y bypass"[Title/Abstract] OR RYGB[Title/Abstract])) |
| --- | --- |
| Web of Science | (ALL=(Postoperative AND (Bleeding OR Hemorrhage OR haemorrhage OR "blood loss")) AND TS=("Gastric bypass"OR "Bariatric bypass surgery"OR "Roux-en-Y bypass"OR RYGB)) |
| Scopus | (ALL ((postoperative AND ( bleeding OR hemorrhage OR haemorrhage OR "blood loss"))) AND TITLE-ABS-KEY (("Gastric bypass" OR "Bariatric bypass surgery" OR "Roux-en-Y bypass" OR rygb ))) |

**Supplementary Table 2** Detailed Inclusion and Exclusion for studies selection

| **Inclusion criteria** | **Exclusion Criteria** |
| --- | --- |
| (i) Indication for RYGB was obesity treatment | (i) Reviews, Case reports, Editorials, Letters to authors, Ecological studies |
| (ii) Adults (age ≥ 18 years) | (ii) Studies in which data/ outcome could not be extracted from the pooled results |
| (iii) Patients with a BMI ≥ 40 kg/m2 or BMI ≥ 35 kg/m2 with weight-related comorbidities. | (iii) Studies in which risk factors for early postoperative bleeding were not analyzed before RYGB |
| (iv) Studies comparing early (< 30 days) postoperative bleeding ª rates in the presence or absence of one or more risk factors | (iv) Studies focus solely on a given preoperative risk factor not viable for analysis |
| (v) If a separate analyses for RYGB was conducted, other types of bariatric surgeries could be included | (v) Analyzed outcomes only in a subset of patients not representative of the general bariatric population |

BMI: Body Index Mass (kg/m^2^); RYGB laparoscopic Roux-en-Y gastric bypass

ᵃ Early postoperative bleeding was defined as bleeding occurring in a period of up to 30 days after RYBG surgery

**Supplementary Table 3** Basic Clinical Characteristics of included studies

| **Study** | **Years of enrollment** | **Country** | **Age (Mean±SD)** | **Gender M/F (n)** | **Type of RYGB (n)** | | | **Type of Surgery (n)** | | **Comorbidities (n)** | | | | | | |
| --- | --- | --- | --- | --- | --- | --- | --- | --- | --- | --- | --- | --- | --- | --- | --- | --- |
|  |  |  |  |  | **ORYGB** | **LRYGB** | **RRYGB** | **Primary** | **Revisional** | **Superobesity (BMI≥50 kg/m2)** | **Diabetes** | **Dyslipidemia** | **Hypertension** | **Cardiovascular Disease** | **GERD** | **OSA** |
| Bakhos C. | 2003-2005 | USA | 44.08 ± 9.99 | 25/107 | 89 | 43 | 0 | 132 | 0 | 63 | NR | NR | NR | NR | NR | NR |
| Dick A. | 2005-2008 | USA | 44.09 ± 11.9 | 140/636 | 131 | 632 | 0 | 770 | 13 | NR | 295 | 307 | 510 | NR | 333 | 270 |
| Rabl C. | 2004-2009 | Austria | 44.08 ± 10.6 | 135/587 | 78 | 644 | 0 | 722 | 0 | NR | 238 | NR | 451 | NR | NR | 255 |
| Deylgat B. | 2003-2009 | Belgium | 39.94 | 201/523 | 16 | 708 | 0 | 652 | 72 | NR | NR | NR | NR | NR | NR | NR |
| Slegtenhorst B.R. | 2007-2009 | Netherlands | 39.99 ± 9.17 | 43/240 | 19 | 264 | 0 | 217 | 66 | NR | 58 | NR | 85 | NR | NR | NR |
| Stenberg E. | 2007-2012 | Sweden | 41.1 ± 10.97 | 6021/19017 | 0 | 25038 | 0 | 25038 | 0 | NR | 3760 | 2540 | 6321 | 150 | NR | 2445 |
| Sadot E. | 2000-2009 | Israel | 42.5 ± 9.28 | 13/113 | 0 | 126 | 0 | 82 | 44 | NR | NR | NR | NR | NR | NR | NR |
| Coblijn U.K. | 2007-2015 | Netherlands | 44.6 ± 14.4 | 296/1371 | NA | 1667 | 0 | 1359 | 308 | NR | 462 | 372 | 669 | NR | NR | 307 |
| Ramly E.P. | 2008-2014 | Lebanon | 45.07 ± 11.62 | 13563/52515 | 0 | 66078 | 0 | 64866 | 1212 | NR | 20927 | NR | 35760 | NR | NR | NR |
| Al-Kurd A. | 2007-2017 | Israel | 43.1 ± 10.9 | 99/223 | NA | 322 | 0 | 161 | 161 | NR | 132 | 125 | 120 | NR | NR | 66 |
| Zafar S.N. | 2015-2017 | USA | 45.4 ± 12 | 8639/34641 | 0 | 43280 | 0 | 39599 | 3681 | 11371 | 28530 | 12674 | 23021 | 390 | 16498 | 17963 |
| Axer S. | 2007-2016 | Sweden | 41 ± 11.09 | 11528/36322 | 19182 | 28668 | 0 | 46055 | 1795 | NR | 6790 | 1624 | 12085 | NR | NR | NR |
| Nasser H. | 2015-2017 | USA | 47.99 ± 19.99 | 1545/10897 | 0 | 11212 | 1230 | 0 | 12442 | NR | 2473 | 2779 | 5476 | 326 | 6032 | 3763 |
| León-Ballesteros G.P | 2004-2019 | Mexico | 40.52 ± 9.62 | 353/496 | 0 | 849 | 0 | 849 | 0 | NR | 241 | 534 | 307 | NR | NR | 58 |
| Poublon N. | 2012-2017 | Netherlands | 48 ± 9.6 | 48/258 | 0 | 306 | 0 | 0 | 306 | NR | 63 | 82 | NR | 96 | 32 | 23 |
| Turchi M.J | 2008-2018 | Argentina | 44.73 ± 5.17 | 165/417 | 0 | 582 | 0 | 582 | 0 | NR | 92 | 380 | 243 | NR | NR | NR |
| Jung J.J | 2015-2017 | USA | 45.85 ± 11.23 | 2435/12461 | 0 | 14896 | 0 | 13756 | 1140 | NR | 5191 | 4167 | 7609 | 514 | 5121 | 5577 |
| Joel S. Frieder | 2010-2018 | USA | 71.7 ± 4.54 | 115/206 | 0 | 321 | 0 | 321 | 0 | NR | 124 | NR | 102 | NR | NR | NR |
| Odovic M. | 1999-2020 | Switzerland | 42.2 ± 11.4 | 634/2005 | 0 | 2639 | 0 | 2386 | 253 | 459 | 1762 | 1778 | 1225 | NR | NR | 1453 |
| Campo-Betancourth C.F | 2008-2021 | Spain | 46.13 | 188/440 | 0 | 628 | 0 | 580 | 48 | NR | 220 | 240 | 302 | 23 | 6 | 402 |
| Pereira A. | 2019 | Portugal | 46 | 38/302 | 0 | 340 | 0 | 257 | 83 | NR | 95 | 154 | 158 | NR | 63 | 58 |
| O’Laughlin M. | 2020 - 2021 | USA | 45.64 ± 10.89 | 6527/10593 | 0 | 11824 | 0 | 5912 | 5912 | NR | 1522 | 2203 | 4469 | 259 | 7917 | 3086 |
| Reiter A.J | 2012-2021 | USA | NR | NR | 0 | 656 | 0 | 656 | 0 | NR | NR | NR | NR | NR | NR | NR |

n: number of patients, M/F: Male/ Female gender, SD: Standard Deviation; ORYGB: laparotomy Roux-en-Y gastric bypass; LRYGB laparoscopic Roux-en-Y gastric bypass; RRYGB: Robotic Roux-en-Y gastric bypass Cardiovascular Disease includes Ischemic heart disease (Acute coronary syndrome, Stable coronary artery disease, history of arterial revascularization), Stroke or Transient Ischemic Attack, Peripheral artery disease, Aortic aneurysm; GERD: Gastroesophageal reflux disease, OSA: Obstructive sleep apnea; NR: Not reported

| **Studies** | **Q1** | **Q2** | **Q3** | **Q4** | **Q5** | **Q6** | **Q7** | **Q8** | **Q9** | **Q10** | **Q11** | **Q12** | **Total MINORS Score** | **Maximum possible score** |
| --- | --- | --- | --- | --- | --- | --- | --- | --- | --- | --- | --- | --- | --- | --- |
| Bakhos C. | 2 | 2 | 0 | 1 | 0 | 2 | 2 | 0 | 2 | 2 | 2 | 2 | 17 | 24 |
| Dick A. | 2 | 2 | 2 | 2 | 0 | 2 | 0 | 0 | 2 | 2 | 2 | 2 | 18 | 24 |
| Rabl C. | 2 | 2 | 0 | 2 | 0 | 2 | 2 | 0 | 2 | 2 | 2 | 0 | 16 | 24 |
| Deylgat B. | 2 | 2 | 0 | 1 | 0 | 2 | 2 | 0 | 1 | 2 | 0 | 0 | 12 | 24 |
| Slegtenhorst B.R. | 2 | 2 | 0 | 2 | 0 | 2 | 2 | 0 | 2 | 2 | 2 | 0 | 16 | 24 |
| Stenberg E. | 2 | 2 | 2 | 2 | 0 | 2 | 2 | 0 |  |  |  |  | 12 | 16 |
| Sadot E. | 2 | 2 | 0 | 2 | 0 | 2 | 1 | 0 | 2 | 2 | 1 | 2 | 16 | 24 |
| Coblijn U.K. | 2 | 2 | 0 | 2 | 0 | 2 | 1 | 0 | 2 | 2 | 1 | 0 | 14 | 24 |
| Ramly E.P. | 2 | 2 | 2 | 2 | 0 | 2 | 0 | 0 | 2 | 2 | 1 | 2 | 17 | 24 |
| Al-Kurd A. | 2 | 2 | 2 | 2 | 0 | 2 | 1 | 0 | 2 | 2 | 2 | 0 | 17 | 24 |
| Zafar S.N. | 2 | 2 | 0 | 2 | 0 | 2 | 0 | 0 | 2 | 2 | 1 | 2 | 15 | 24 |
| Axer S. | 2 | 2 | 0 | 2 | 0 | 2 | 0 | 0 | 2 | 2 | 1 | 2 | 15 | 24 |
| Nasser H. | 2 | 2 | 0 | 2 | 0 | 2 | 0 | 0 |  |  |  |  | 8 | 16 |
| León-Ballesteros G.P | 2 | 2 | 2 | 2 | 0 | 2 | 1 | 0 | 2 | 2 | 1 | 2 | 18 | 24 |
| Poublon N. | 2 | 2 | 0 | 2 | 0 | 2 | 0 | 0 |  |  |  |  | 8 | 16 |
| Turchi M.J | 2 | 2 | 0 | 2 | 0 | 2 | 0 | 0 | 2 | 2 | 2 | 2 | 16 | 24 |
| Jung J.J | 2 | 2 | 0 | 2 | 0 | 2 | 2 | 0 | 2 | 2 | 2 | 2 | 18 | 24 |
| Joel S. Frieder | 2 | 2 | 0 | 1 | 0 | 2 | 1 | 0 | 2 | 2 | 0 | 2 | 14 | 24 |
| Odovic M. | 2 | 2 | 0 | 2 | 0 | 2 | 2 | 0 | 2 | 2 | 2 | 2 | 18 | 24 |
| Campo-Betancourth C.F | 2 | 2 | 0 | 1 | 0 | 2 | 0 | 0 | 2 | 2 | 1 | 0 | 12 | 24 |
| Pereira A. | 2 | 2 | 0 | 2 | 0 | 2 | 2 | 0 | 2 | 2 | 2 | 2 | 18 | 24 |
| O’Laughlin M. | 2 | 2 | 0 | 2 | 0 | 2 | 0 | 0 | 2 | 2 | 2 | 2 | 16 | 24 |
| Reiter A.J | 2 | 2 | 0 | 2 | 0 | 2 | 0 | 0 | 2 | 2 | 2 | 2 | 16 | 24 |

**Supplementary Table 4** Detailed results of the risk of bias assessments for included primary studies using MINORS risk of bias

The items are scored 0 (not reported), 1 (reported but inadequate) or 2 (reported and adequate). The global ideal score is 16 for non-comparative studies and 24 for comparative studies.

Q1. A clearly stated aim: the question addressed should be precise and relevant in the light of available literature

Q2. Inclusion of consecutive patients: all patients potentially fit for inclusion (satisfying the criteria for inclusion) have been included in the study during the study period (no exclusion or details about the reasons for exclusion)

Q3. Prospective collection of data: data were collected according to a protocol established before the beginning of the study

Q4. Endpoints appropriate to the aim of the study: unambiguous explanation of the criteria used to evaluate the main outcome which should be in accordance with the question addressed by the study. Also, the endpoints should be assessed on an intention-to-treat basis.

Q5. Unbiased assessment of the study endpoint: blind evaluation of objective endpoints and double-blind evaluation of subjective endpoints. Otherwise the reasons for not blinding should be stated

Q6. Follow-up period appropriate to the aim of the study: the follow-up should be sufficiently long to allow the assessment of

the main endpoint and possible adverse events

Q7. Loss to follow-up less than 5%: all patients should be included in the follow-up. Otherwise, the proportion lost to follow-up should not exceed the proportion experiencing the major endpoint

Q8. Prospective calculation of the study size: information on the size of detectable difference of interest with a calculation of 95% confidence interval, according to the expected incidence of the outcome event, and information about the level for statistical significance and estimates of power when comparing the outcomes

Q9. An adequate control group: having a gold standard diagnostic test or therapeutic intervention recognized as the optimal intervention according to the available published data

Q10. Contemporary groups: control and studied groups should be managed during the same time period (no historical comparison)

Q11. Baseline equivalence of groups: the groups should be similar regarding the criteria other than the studied endpoints. Absence of confounding factors that could bias the interpretation of the results

Q12. Adequate statistical analyses: whether the statistics were in accordance with the type of study with the calculation of confidence intervals or relative risk

**Supplementary Table 5** Leave-one-out sensitivity analyses for Age in early postoperative bleeding after RYGB

| **Omitted Study** | **MD, 95% CI, p-value** | **I2** |
| --- | --- | --- |
| Bakhos C., 2009 | 2.17; [0.031-4.31]; (0.0467) | 0.0% |
| Dick A., 2010 | 2.75; [0.44-5.05]; (0.0196) | 21.7% |
| Rabl C., 2011 | 3.43; [1.39-5.48]; (0.0010) | 0.0% |
| Odovic M., 2022 | 2.77; [0.046-5.50]; (0.0463) | 21.9% |

**Supplementary Table 6** Leave-one-out sensitivity analyses for Gender in early postoperative bleeding after RYGB

| **Omitted Study** | **RR, 95% CI, p-value** | **I^2^** |
| --- | --- | --- |
| Bakhos C., 2009 | 1.44; [1.20; 1.72]; (< 0.0001) | 28.4% |
| Dick A., 2010 | 1.41; [1.18-1.70]; (0.0002) | 30.9% |
| Rabl C., 2011 | 1.43; [1.27-1.61]; (< 0.0001) | 0.0% |
| Stenberg E., 2014 | 1.30; [1.11-1.53]; (0.0010) | 0.0% |
| Zafar SN., 2018 | 1.55; [1.32-1.82]; (< 0.0001) | 0.0% |
| Odovic M., 2022 | 1.39; [1.15-1.68]; (0.0007) | 27.2% |
| Pereira A., 2022 | 1.41; [1.18-1.67]; (0.0001) | 26.9% |

**Supplementary Table 7** Leave-one-out sensitivity analyses for BMI > 50 kg/m^2^ (Superobesity) in early postoperative bleeding after RYGB

| **Omitted Study** | **RR, 95% CI, p-value** | **I^2^** |
| --- | --- | --- |
| Bakhos C., 2009 | 0.98; [0.83-1.16]; (0.8133) | 0.0% |
| Zafar SN., 2018 | 0.84; [0.54-1.31]; (0.4513) | 0.0% |
| Odovic M., 2022 | 0.99; [0.84; 1.17; (0.9049) | 0.0% |

**Supplementary Table 8** Leave-one-out sensitivity analyses for Diabetes Mellitus in early postoperative bleeding after RYGB

| **Omitted Study** | **RR, 95% CI, p-value** | **I^2^** |
| --- | --- | --- |
| Dick A., 2010 | 1.24 [0.76-2.01]; (0.3891) | 89.5% |
| Rabal C., 2011 | 1.00 [0.63-1.56]; (0.9881) | 88.6% |
| Stenberg E., 2014 | 0.95 [0.66-1.38]; (0.8038) | 50.8% |
| Zafar SN., 2018 | 1.26 [0.80-2.00]; (0.3140) | 65.7% |
| Odovic M., 2022 | 1.18 [0.69-2.01]; (0.5399) | 89.9% |
| Pereira A., 2022 | 1.14 [0.71-1.84]; (0.5925) | 89.9% |

**Supplementary Table 9** Leave-one-out sensitivity analyses for Hypertension in early postoperative bleeding after RYGB

| **Omitted Study** | **RR, 95% CI, p-value** | **I^2^** |
| --- | --- | --- |
| Dick A., 2010 | 1.50 [1.23-1.83]; (< 0.0001) | 46.4% |
| Rabl C., 2011 | 1.35 [1.02-1.79]; (0.0386) | 71.5% |
| Stenberg E., 2014 | 1.13 [0.78-1.64]; (0.5118) | 55.6% |
| Zafar SN., 2018 | 1.17 [0.74-1.84]; (0.6027) | 69.0% |
| Odovic M., 2022 | 1.24 [0.90-1.69]; (0.1846) | 72.0% |
| Pereira A., 2022 | 1.39 [1.07-1.81]; (0.0125) | 67.3% |

**Supplementary Table 10** Leave-one-out sensitivity analyses for Dyslipidemia in early postoperative bleeding after RYGB

| **Omitted Study** | **RR, 95% CI, p-value** | **I^2^** |
| --- | --- | --- |
| Dick A., 2010 | 1.21; [0.85-1.72]; (0.2823) | 78.3% |
| Stenberg E., 2014 | 0.85; [0.52-1.38]; (0.5080) | 69.8% |
| Zafar SN., 2018 | 0.88; [0.44-1.77]; (0.7181) | 84.3% |
| Odovic M., 2022 | 1.20; [0.82-1.77]; (0.3463) | 77.3% |
| Pereira A., 2022 | 1.12 [0.76-1.65]; (0.5594) | 83.0% |

| **Omitted Study** | **RR, 95% CI, p-value** | **I^2^** |
| --- | --- | --- |
| Dick A., 2010 | 1.15 [0.91-1.44]; (0.2438) | 44.5% |
| Rabal C., 2011 | 1.17 [0.96-1.43]; (0.1136) | 34.0% |
| Stenberg E., 2014 | 1.07 [0.93-1.24]; (0.3265) | 0.0% |
| Zafar SN., 2018 | 1.10 [0.78-1.55]; (0.5709) | 36.9% |
| Odovic M., 2022 | 1.17 [0.92-1.49]; (0.1888) | 39.8% |
| Pereira A., 2022 | 1.16 [0.96-1.41]; (0.1311) | 34.4% |

**Supplementary Table 11** Leave-one-out sensitivity analyses for Obstructive Sleep Apnea in early postoperative bleeding after RYGB

**Supplementary Table 12** Leave-one-out sensitivity analyses for GERD in early postoperative bleeding after RYGB

| **Omitted Study** | **RR, 95% CI, p-value** | **I^2^** |
| --- | --- | --- |
| Dick A., 2010 | 1.40; [0.79-2.49]; (0.2519) | 41.2% |
| Zafar SN., 2018 | 1.26; [0.38-4.20]; (0.7112) | 70.2% |
| Odovic M., 2022 | 1.08; [0.72-1.62]; (0.7115) | 35.9% |

**Supplementary Table 13** Leave-one-out sensitivity analyses for Revisional Bariatric Surgery in early postoperative bleeding after RYGB

| **Omitted Study** | **RR, 95% CI, p-value** | **I^2^** |
| --- | --- | --- |
| Dick A., 2010 | 1.33 [1.10-1.62]; (0.0034) | 28.1% |
| Deylgat B., 2012 | 1.34 [1.12-1.60]; (0.0015) | 19.8% |
| Slegtenhorst BR., 2013 | 1.34 [1.10-1.62]; (0.0032) | 28.0% |
| Sadot E., 2015 | 1.34 [1.10-1.63]; (0.0031) | 27.6% |
| Coblijn UK., 2016 | 1.33 [1.09-1.63]; (0.0052) | 28.0% |
| Ramly EP., 2016 | 1.37 [1.12-1.67]; (0.0021) | 23.7% |
| Al-Kurd A., 2018 | 1.41 [1.20-1.66]; (<0.0001) | 11.5% |
| Zafar SN., 2018 | 1.27 [1.07-1.52]; (0.0077) | 2.6% |
| Axer S., 2019 | 1.29 [1.02-1.62]; (0.0341) | 28.4% |
| Jung JJ., 2021 | 1.30 [1.05-1.61]: (0.0160) | 28.0% |
| Odovic M., 2022 | 1.40 [1.18-1.66]; (0.0001) | 15.3% |
| Pereira A., 2022 | 1.33 [1.09-1.61]; (0.0048) | 27.6% |
| Campo-Betancourth CF., 2022 | 1.41 [1.20-1.64]; (< 0.0001) | 10.1% |
| O’Laughlin M., 2023 | 1.42 [1.18-1.69]; (0.0002) | 16.2% |

**Supplementary Figure Legends**

**Supplementary Figure 1** - Forest plot representing the mean difference in age (measured in years) between bleeding and not-bleeding patients. (mean difference with 95% CI – random effects meta-analysis).

**Supplementary Figure 2** - Forest plot representing the effect of male gender compared to female gender in early postoperative bleeding after RYGB (RR with 95% CI – random effects meta-analysis).

**Supplementary Figure 3** - Forest plot representing the effect of preoperative BMI > 50 kg/m^2^ compared with BMI < 50 kg/m^2^ in early postoperative bleeding after RYGB (RR with 95% CI – random effects meta-analysis).

**Supplementary Figure 4** - Forest plot representing the effect of preoperative diabetes mellitus compared with the absence of a preoperative diagnosis of diabetes mellitus in early postoperative bleeding after RYGB (RR with 95% CI – random effects meta-analysis).

**Supplementary Figure 5** - Forest plot representing the effect of preoperative arterial hypertension (HTN) compared with the absence of a preoperative diagnosis of arterial hypertension in early postoperative bleeding after RYGB (RR with 95% CI – random effects meta-analysis).

**Supplementary Figure 6** - Forest plot representing the effect of preoperative dyslipidemia compared with the absence of a preoperative diagnosis of dyslipidemia in early postoperative bleeding after RYGB (RR with 95% CI – random effects meta-analysis).

**Supplementary Figure 7** - Forest plot representing the effect of preoperative obstructive sleep apnea (OSA) compared with the absence of a preoperative diagnosis of OSA in early postoperative bleeding after RYGB (RR with 95% CI – random effects meta-analysis).

**Supplementary Figure 8** - Forest plot representing the effect of preoperative gastroesophageal reflux disease (GERD) compared with the absence of a preoperative diagnosis of GERD in early postoperative bleeding after RYGB (RR with 95% CI – random effects meta-analysis).

**Supplementary Figure 9** - Forest plot representing the effect of revisional RYGB surgery (rRYGB) compared with primary RYGB surgery (pRYGB) in early postoperative bleeding after RYGB (RR with 95% CI – random effects meta-analysis).

**Supplementary Figure 10** - Forest plot representing the effect of prophylaxis for thromboembolism with unfractionated heparin compared with use of enoxaparin in early postoperative bleeding after RYGB (RR with 95% CI – random effects meta-analysis).

**Supplementary
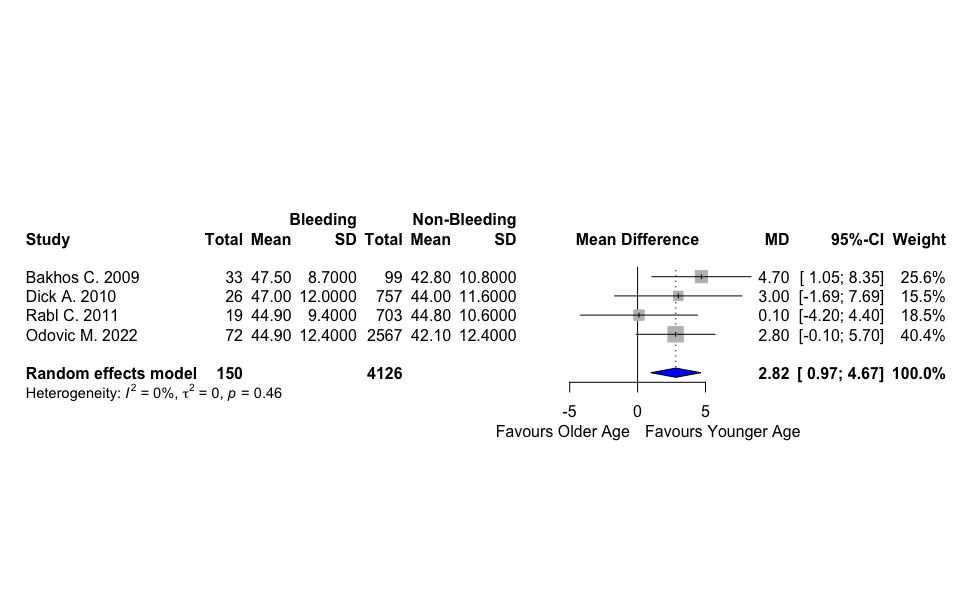
Figure 1**

**Supplementary Figure 2**

**
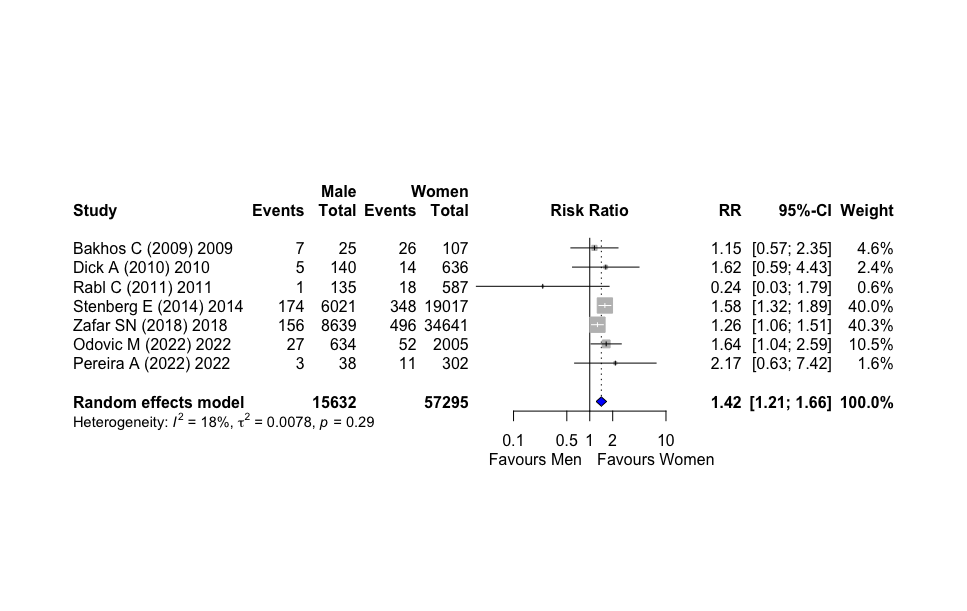
**

**Supplementary
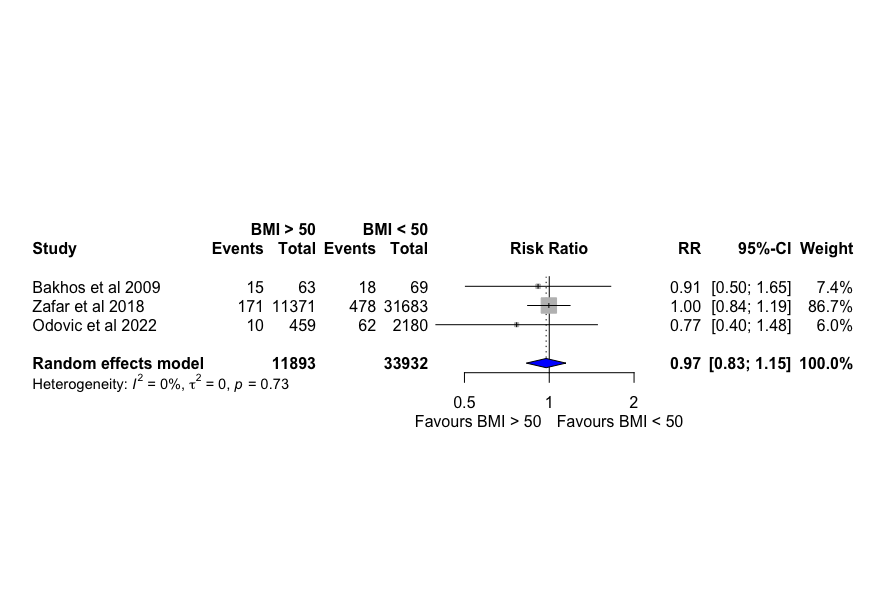
Figure 3**

**
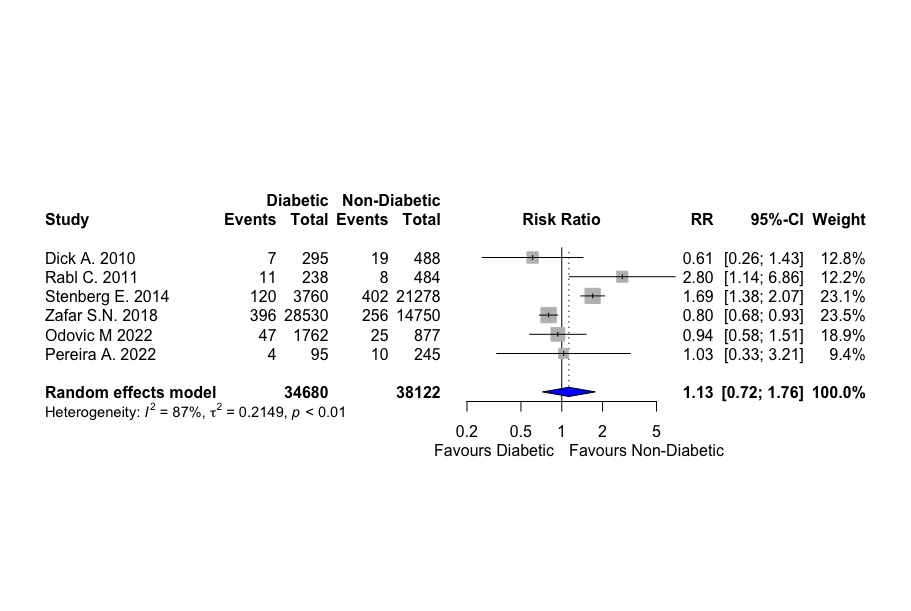
Supplementary Figure 4**

**Supplementary Figure 5**

**
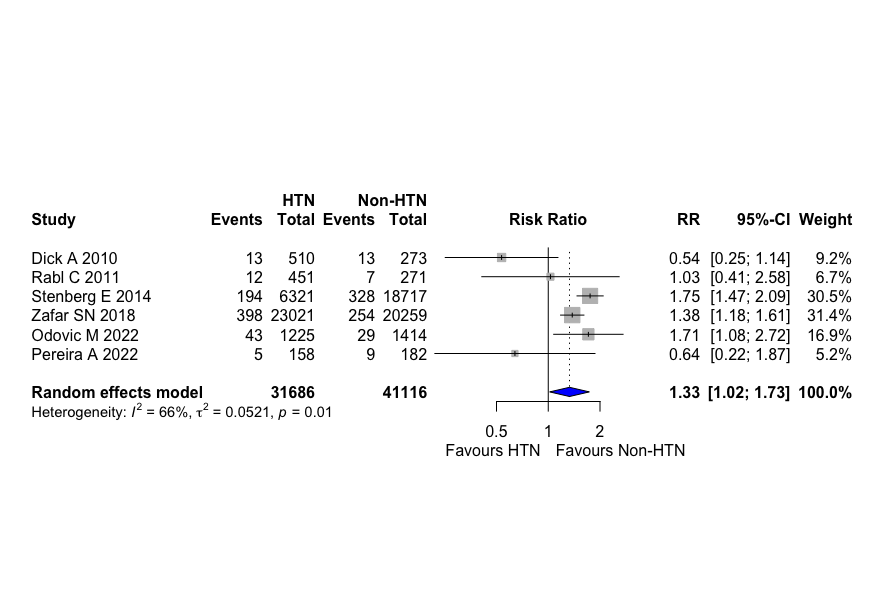
**

**
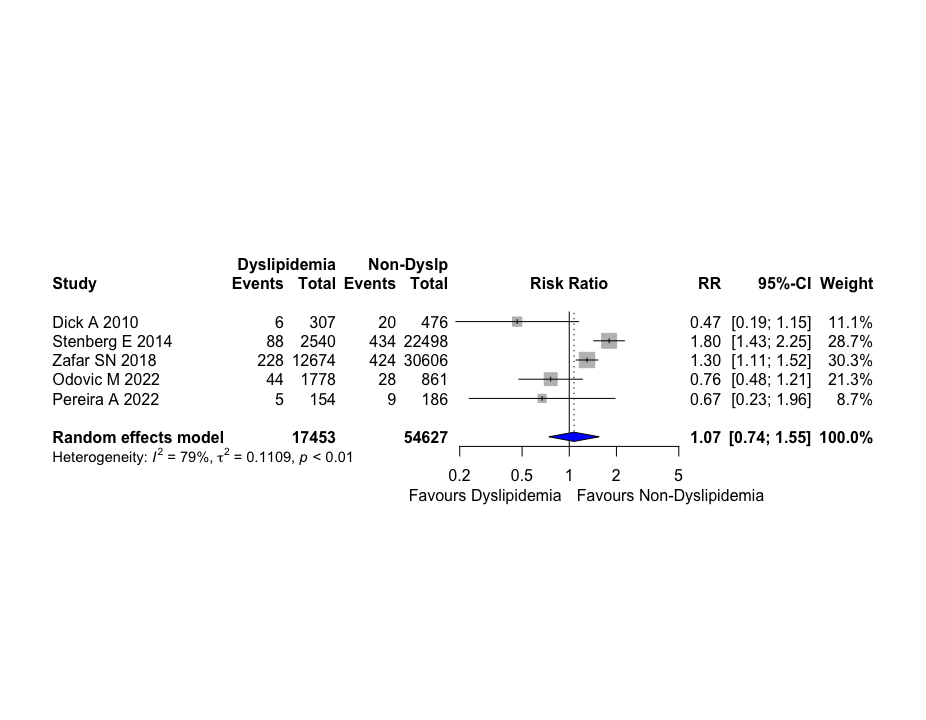
Supplementary Figure 6**

**Supplementary
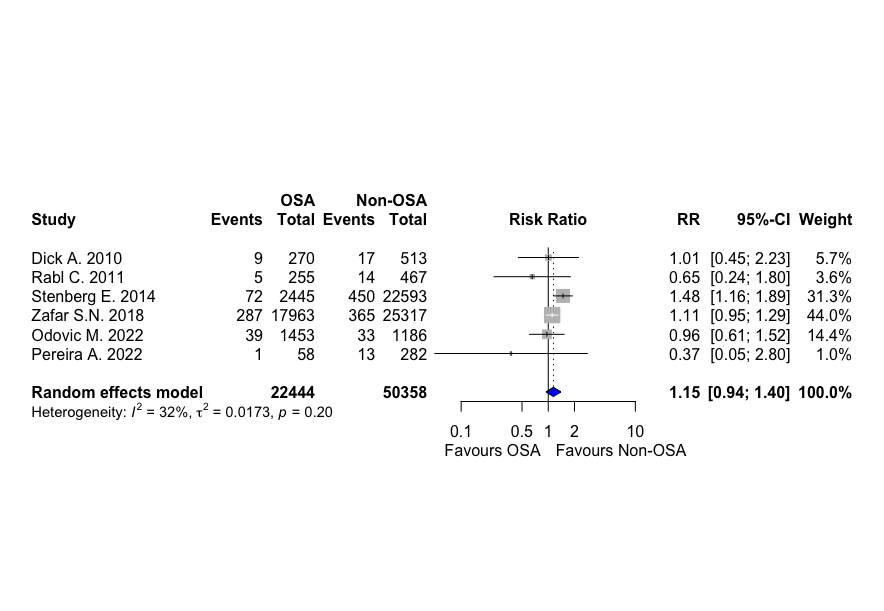
Figure 7**

**Supplementary Figure 8**

**
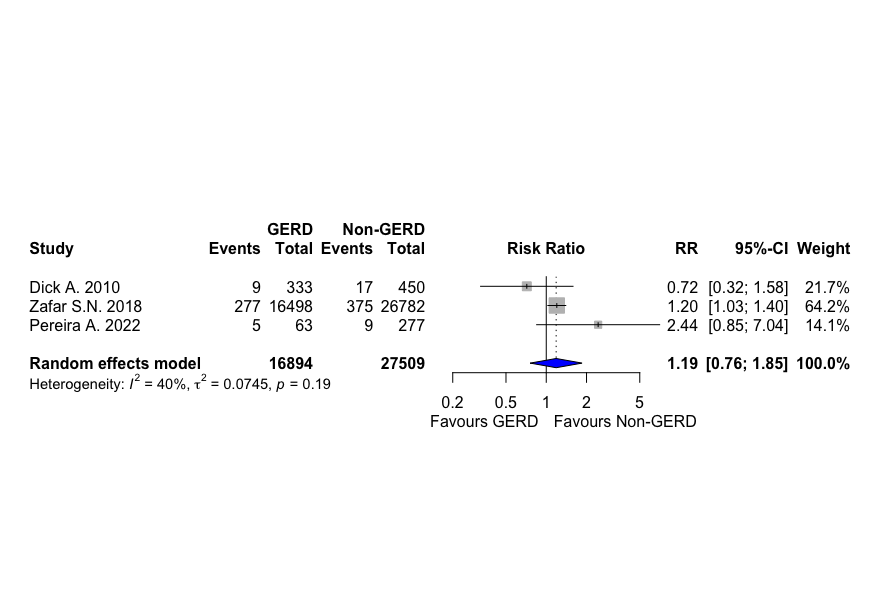
**

**Supplementary Figure 9**

**
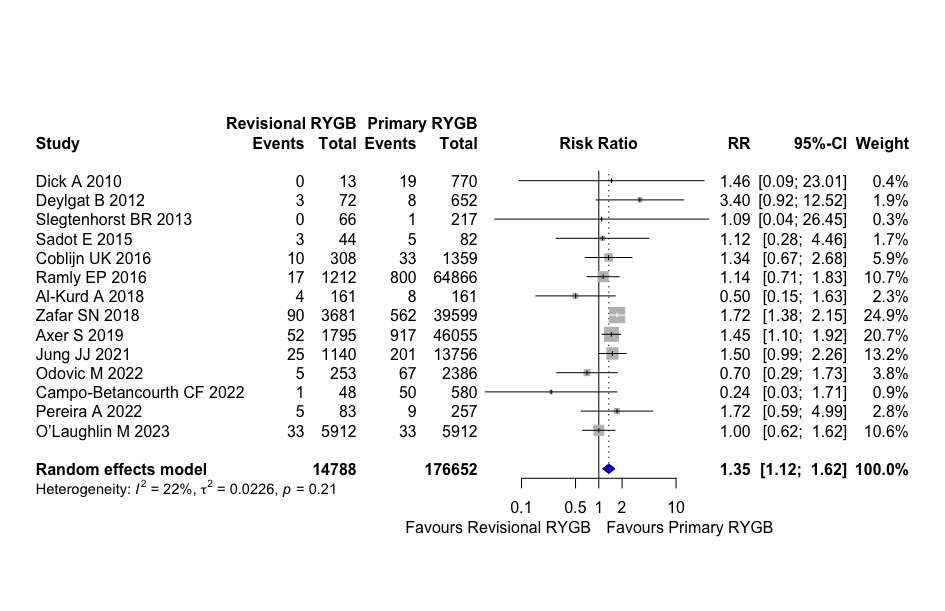
 Supplementary Figure 10**


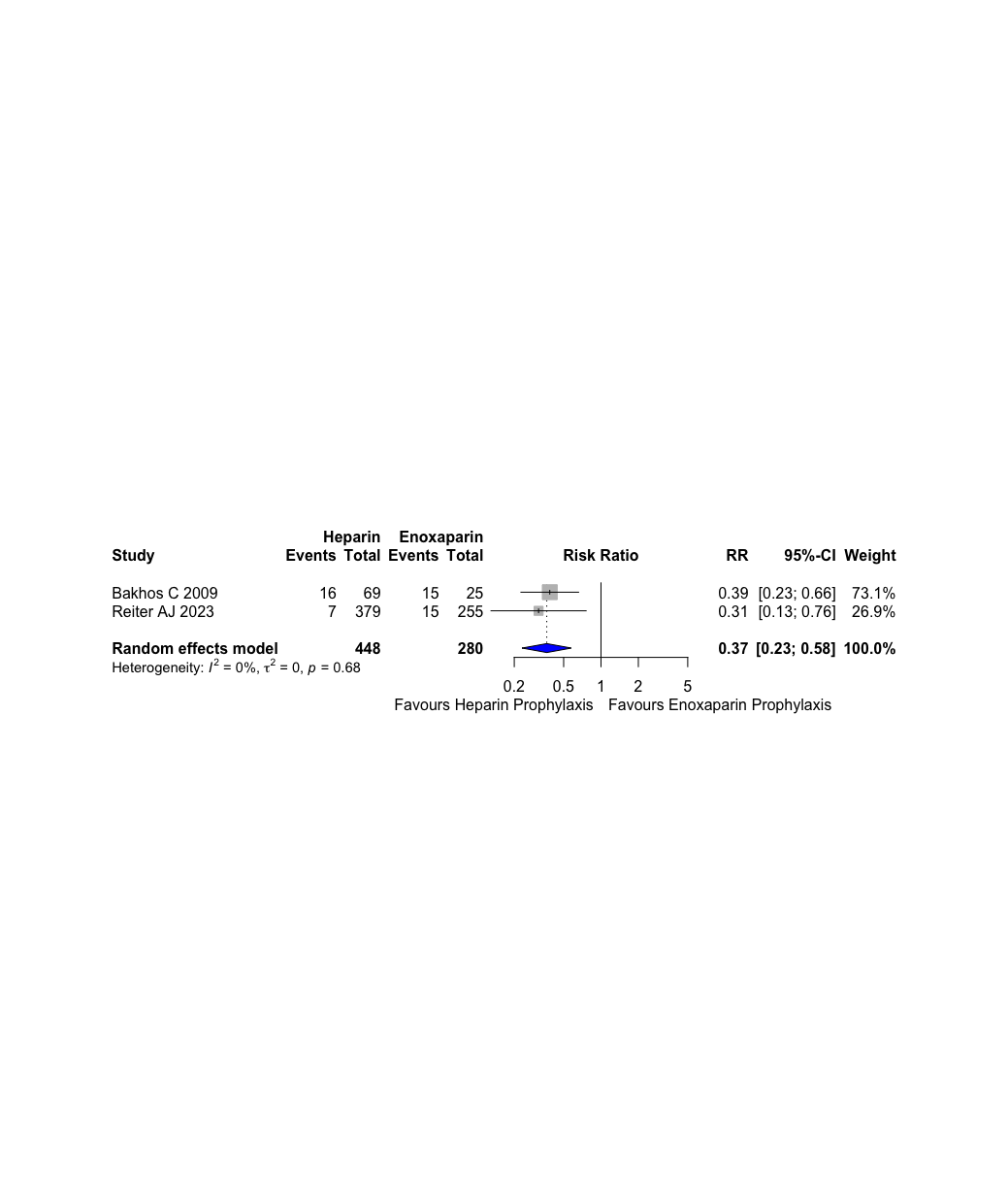

Supplement: Supplementary file 1 — Supplementary Material 1 [file 423_2024_3346_MOESM1_ESM.docx]
